# From Cell Entry to Engraftment of Exogenous Mitochondria

**DOI:** 10.3390/ijms21144995

**Published:** 2020-07-15

**Authors:** Daisuke Kami, Satoshi Gojo

**Affiliations:** Department of Regenerative Medicine, Kyoto Prefectural University of Medicine, Kyoto 6028566, Japan; dkami@koto.kpu-m.ac.jp

**Keywords:** mitochondria, macropinocytosis, exogenous mitochondrial transfer

## Abstract

Mitochondrial transfer has been recognized to play a role in a variety of processes, ranging from fertilization to cancer and neurodegenerative diseases as well as mammalian horizontal gene transfer. It is achieved through either exogeneous or intercellular mitochondrial transfer. From the viewpoint of evolution, exogeneous mitochondrial transfer is quite akin to the initial process of symbiosis between α-protobacterium and archaea, although the progeny have developed more sophisticated machinery to engulf environmental materials, including nutrients, bacteria, and viruses. A molecular-based knowledge of endocytosis, including macropinocytosis and endosomal escape involving bacteria and viruses, could provide mechanistic insights into exogeneous mitochondrial transfer. We focus on exogeneous mitochondrial transfer in this review to facilitate the clinical development of the use of isolated mitochondria to treat various pathological conditions. Several kinds of novel procedures to enhance exogeneous mitochondrial transfer have been developed and are summarized in this review.

## 1. Introduction

Mitochondria are considered to be derived from α-protobacterium that entered and symbiotically dwelled in archaea [1,2]. Upon entry, the α-protobacterium disrupted the target archaea, which protected themselves against the α-protobacterium. The progenies of archaea that developed a symbiosis with the α-protobacterium evolved into eukaryotes, leaving behind vestiges that we can observe through nutrient uptake and bacterial or viral infections [3]. Exogeneous isolated mitochondria are engulfed into recipient cells that are in proximity by macropinocytosis [4]. The α-protobacteria might also take advantage of macropinocytosis for destination, whereas archaea might engulf α-protobacteria along with nutrient acquisition by macropinocytosis. Alternatively, an interaction between the two prior to passing across the plasma membrane might induce endocytosis, including macropinocytosis. Macropinocytosis is regulated by the sensing of environmental cues [5], and macropinosomes that are pinched off the plasma membrane might be leaky or fragile enough for the cargo to be liberated into the cytosol, as it has been reported from independent researchers that exogenously isolated mitochondria complement the respiratory deficits in host cells [4,6]. Currently, mitochondrial transfer that is mediated by either exogeneous [7,8,9,10] or intercellular transfer [11] is ensured not only in vitro but also in vivo in pathophysiological situations, ranging from fertilization [12] to cancer [13] and neurodegenerative diseases [14]. In particular, exogeneous mitochondrial transplantation has been intensively investigated in animal models of various diseases, including neurological and cardiovascular diseases, and major related reports are summarized in Table 1. Neurons suffering from in vivo ischemia-reperfusion injuries showed enhanced expression of survival-related genes such as BCL-XL and suppressed expression of apoptosis-related genes such as caspase 3 after direct injection of astrocyte-derived mitochondrial particles into the IR-damaged cortex [15]. Mitochondrial transfer was reported to rescue IR damage through either intracerebral or intra-arterial injection, resulting in restored motor performance with attenuated cell death [16]. Although treatment of spinal cord injury with exogeneous mitochondrial transfer showed conservation of bioenergetics in the short-term, the effects were not maintained long-term [17]. Similar to the effects on IR damage in the brain, IR injuries to heart were also improved by exogeneous mitochondrial transfer [18]. In the liver after IR injury, mitochondrial transfer suppressed hepatic cell death [19]. Similar effects were observed in IR-damaged lungs after either intra-arterial or trans-tracheal delivery of exogeneous mitochondria [20]. Regardless of the organ, IR injuries appear to be a promising clinical target of exogeneous mitochondrial transfer, as independent researchers have achieved functional rescue, despite differences in efficacy among reports. However, mechanistic approaches for the examination of the process of exogeneous transfer have been more scarce than those for the process of intercellular transfer. Although the way in which the trajectory of exogeneous mitochondria from outside the cell to the final destination inside the host cell is achieved still remains to be elucidated in terms of molecular aspects, virus entry could offer us clues for better understanding of the molecular regulation involved in mitochondrial transfer. In this review, we focus on molecular mechanistic insights into the internalization of exogeneous materials, nutrients, or viruses to provide viewpoints to reveal the mechanism of mitochondrial transfer. In addition, we reviewed the current technology that is used to enhance exogeneous mitochondrial transfer.

## 2. Cell Entry

Isolated mitochondria are mainly engulfed via macropinocytosis [4], although other processes are involved in the internalization of exogeneous mitochondria. Macropinocytosis is a process in which eukaryotes engulf extracellular materials, including fluids, and it is well conserved from amoebae to humans, suggesting that macropinocytosis could have been acquired at an early stage of evolution [21] and might have been involved in the first occurrence of symbiosis. Macropinocytosis is a form of pinocytosis, which does not depend upon dynamin, whereas phagocytosis, another form of endocytosis, involves the ingestion of large particles, including solid objects [22]. The two processes share common features, such as a large vacuole size, transient activation, and actin dependence, but are different in several aspects. Phagocytosis requires receptor interaction with the particle for ingestion, involves the uptake of a limited fluid volume due to the tight wrapping of the particle, is specific for the particle in specialized cells, such as macrophages, and activates localized actin modifications. On the other hand, macropinocytosis does not require the particle to interact with receptors in plasma membranes, engulfs extracellular fluids, is executed in a nonspecific manner in all cells, and globally causes the formation of either ruffles, lamellipodia, or blebbing over the entire surface of the plasma membrane.

### 2.1. Passage through the Plasma Membrane of Viruses

Virus entry comprises several processes by which the viral genome is transported to the replication site, such as the replication organelle for many minus-stranded RNA viruses. All events, including attachment to the plasma membrane, penetration or endocytosis, endosomal escape, and uncoating, are achieved through the sophisticated collaboration of virus-encoded proteins with the host machinery.

Once the surface receptors on the plasma membrane interact with virions, they convey forward signals into the cells. The battle between the host cell and the virus commences. For example, following the dual attachment of adenoviruses to the plasma membrane via the coxsackievirus adenovirus receptor (CAR), which induces actomyosin-dependent movement of virions, and αvβ3/5 integrins that are static and confined to a given position for virions, the opposing force by the host cell causes the virions to loosen their structures [23], resulting in fiber shedding to reduce avidity [24]. Taking advantage of this process, adenoviruses expose protein VI, which is shielded inside the virion, to generate pores in the plasma membrane, whereas the host secretes acid sphingomyelinase from lysosomes in response to Ca^2+^ influx from the pores to repair the disturbed membrane, which is exploited to facilitate viral endocytosis, including incremental macropinocytosis of ceramide lipids [25]. Although the virus trajectories on the plasma membranes, including directed motion and diffusive motion, exhibit the general features of the virus–host interaction that are driven by the intrinsic properties of the plasma membrane rather than those of the viruses [26], viruses transform the host defensive machinery for the purpose of their own offensive modalities. Unlike viruses, the progenitors of mitochondria could survive without parasitizing cells, so the invasion of cells by mitochondria might not have occurred. However, the components of mitochondria, including mitochondrial DNA, *N*-formyl peptides, and cardiolipin, are strongly immunogenic, meaning that mitochondria are nonself for host cells. Thus, both at the initial occurrence of symbiosis and upon the internalization of exogeneous mitochondria, offense and defense measures should occur.

**Figure 1:**
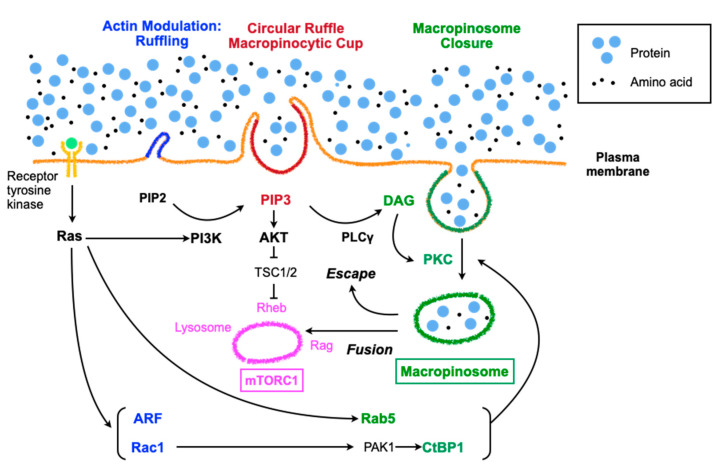
Regulation of macropinocytosis and the signaling pathways involving in mTORC share many molecules, but these molecules do not always function in parallel under various environmental stress conditions. PI3K: phosphoinositide 3-kinase, PIP2: phosphatidylinositol-4, 5-bisphosphate, TSC: tuberous sclerosis complex, DAG: diacylglycerol, PKC: protein kinase C, PLC: phospholipase C.

Most viruses exploit the endocytic activities of the host cell upon entry into the cytoplasm. For example, B-species human adenoviruses bind to CD46 through their fiber knobs [27] and to αvβ3/5 integrins via the viral penton base with different affinities [26], leading to CD46 oligomerization, which triggers macropinocytosis [28]. The signals for macropinocytosis are generally mediated by the Rac-Pak1-CtBP1 axis [29] (Figure 1). Rac, which is downstream of several receptors, including receptor of tyrosine kinases, activates actin polymerization, which is indispensable for membrane ruffling in macropinocytosis and relays signals to Pak1; CtBP1, which is phosphorylated by Pak1, is incorporated into macropinosomes along with Pak1 and suppresses innate immune response via transcriptional repression [30,31]. Similar to adenoviruses, vaccinia virus, coxsackievirus B, herpes simplex virus 1, echovirus 1, and mimivirus are also ingested through macropinocytosis [22]. They initially activate signaling pathways to elicit either ruffles or blebbing. Vaccinia virus elicits a macropinocytotic response characterized by blebbing rather than ruffles and lamellipodia, which seems to mimic the internalization of apoptotic bodies [32]. The membranes of mature virions possess large amounts of phosphatidylserine, which is required for macropinocytosis of apoptotic bodies. Ingestion of extracellular materials seems to occur through the bleb retraction process. This type of macropinocytosis is exploited by the bacterial pathogen *Shigella flexneri* [33]. Poxvirus infection, which mainly utilizes macropinocytosis as a cell entry method [32], rapidly provokes AMPK activation and vice versa, suggesting the involvement of macropinocytosis via AMPK activation upon viral entry [34]. Ebola virus takes advantage of the AMPK signaling pathway to infect cells via macropinocytotic internalization [35]. AMPK phosphorylates the anti-capping protein of actin, vasodilator-stimulated phosphoprotein (VASP), which subsequently promotes actin capping and results in increased cell motility, cytoskeletal rearrangement and reorganization, and the formation of lamellipodia. In addition to the large size of the materials, immune evasion might be another reason that viruses exploit macropinocytosis. Macropinocytosis of apoptotic cells can suppress innate immunity instead of inducing inflammatory responses [36]. This property might work in favor of the ingestion of exogeneous mitochondria by macropinocytosis.

### 2.2. Molecular Aspects of Macropinocytosis

The induction of macropinocytosis takes place in response to growth factors, pathogens, including viruses or bacteria, or apoptotic bodies [22]. Macropinocytosis is an actin-driven process involving rearrangements of filamentous F-actin, which is preceded by the establishment of membrane patches primed for macropinocytosis with high levels of Ras activity and phosphatidylinositol (3,4,5)-triphosphate (PIP3) accumulation [21]. Growth factor-dependent macropinocytosis is activated by receptor tyrosine kinases (RTKs), which relay signals to Ras superfamily GTPases and then initiate parallel signaling pathways involving Rac1, Rab5, Arf6, and PI3K (Figure 1) [22]. Rac1 and Arf6 are involved in actin modulation, whereas Rab5 and CtBP1, which are downstream of Rac1, contribute to macropinosome closure; PI3K mainly regulates macropinocytic cup formation and cup closure. Growth factor-dependent macropinocytosis supplies amino acids to lysosomes, resulting in Rag activation (vesicular pathway), whereas the PI3K-PIP3-AKT pathway leads to Rheb activation (cytosolic pathway), subsequently effectively activating mTORC1 [5]. On the other hand, growth factor-independent macropinocytosis does not involve RTKs but takes advantage of downstream RTKs, especially PI3K and PKC, which are augmented by PI3K, leading to mTORC1 activation (Figure 1). Signaling involved in amino acid detection by a mammalian cell converges on mTORC1, which regulates growth and responses against stresses, including starvation and hypoxia [37]. Macropinocytosis and mTORC1 seem to coordinately contribute to cellular growth by sharing some signaling pathways [5].

In the case of macrophages treated with macrophage colony-stimulating factor (M-CSF), the beginning of macropinocytosis involves the formation of a ruffle-like C-shape, which then forms a loop called a ruffle closure when activated Rac1 accumulates within the cup-like structure surrounded by the ruffle [38]. As the ruffle grows, PI3K is activated and generates PIP3 inside of the cup. Actin polymerization in the ruffles is executed through the recruitment of myosin-I motor proteins and myosin-IB by PIP3 [39]. Other proteins that are recruited by PIP3 are a subset of pleckstrin homology (PH) domain-containing signaling proteins, such as AKT and PDK1 [40]. A well-examined pathway downstream of PIP3 involves phospholipase C-γ (PLCγ), which is phosphorylated and activated by RTKs through the SH2 domain and generates inositol 1,4,5-trisphosphate (IP3) and diacylglycerol (DAG) from PIP2. DAG inside the macropinocytic cup recruits and activates protein kinase C (PKC) [41], which contributes to cup closure at the inlet hole by folding back into the center of the cup and generating macropinosomes to be released into the cytoplasm [42].

### 2.3. Regulation of Macropinocytosis

**Figure 2:**
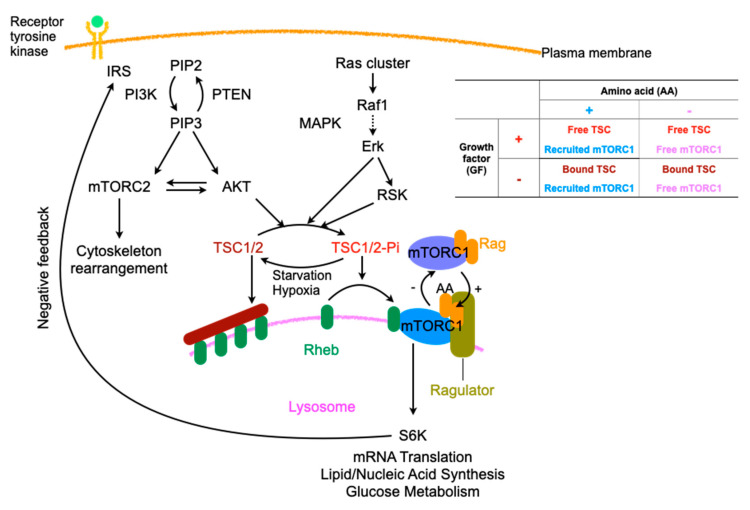
Regulation of mTORC, especially related to amino acids and signals of growth factors. IRS: insulin receptor substrate, PI3K: phosphoinositide 3-kinase, PIP2: phosphatidylinositol-4, 5-bisphosphate, PETN: phosphatase and tensin homolog, mTORC: mTOR complex, MAPK: Mitogen-activated Protein Kinase, RSK: ribosomal protein S6 kinase, TSC: tuberous sclerosis complex, Dotted arrow; abridged signaling factors, In table, +: presence, −: absence.

Growth factors, such as insulin and epidermal growth factor, and extracellular nutrients regulate cell growth and survival. Depending upon these inputs, cells execute either anabolic processes that cause growth or catabolic processes to ensure survival through mechanistic target of rapamycin (mTOR), consisting of mTORC1, which is involved in protein, lipid, and nucleotide synthesis, and mTORC2, which is involved in cytoskeleton rearrangement and glucose metabolism [43]. Ras, mutant variants of which are involved in various cancers, localizes in the membranous portions, including the plasma membrane, where it forms nanoclusters, endoplasmic reticulum, and the Golgi apparatus through its prenylation and palmitoylation. Ras activates macropinocytosis through several signaling cascades, such as the mitogen-activated protein (MAP) kinase and PI3K cascades [44]. Ras is a single-subunit small GTPase that plays the role of a signaling switch that is on when bound to GTP and off when bound to GDP and activates downstream effectors with high affinity binding. The manner of transmission does not involve allosteric changes but rather the release of the autoinhibition of MAPK and the restraint of PI3K on the membrane [45]. PI3K is recruited and allosterically activated by various growth factors and cytokines, such as insulin and epidermal growth factor, resulting in the phosphorylation of phosphatidylinositol (4,5)-bisphosphate (PIP2) to generate PIP3, which in turn recruits and activates effectors with pleckstrin homology (PH) domains such as protein kinase B (also called AKT). AKT amplifies the signals of RTKs through a positive feedback loop with mTORC2 [46]. The growth factor signals converge at and are coordinated by mTORC1, which increase anabolism and suppress catabolism [43]. Both ERK and its effector p90RSK in the MAPK pathway [47] and AKT in the PI3K pathway [48] phosphorylate tuberous sclerosis complex 2 (TSC2), which comprises TSC along with TSC1 and TBC1D7, an upstream inhibitor of mTORC1 [49], via the suppression of Ras homolog enriched in the brain (Rheb); Rheb is an immediate upstream activator of mTORC1 and is constitutively present on the lysosomal surface [50]. The phosphorylation of TSC by AKT induces its dissociation from the lysosomal membrane rather than a reduction in intrinsic TSC2 GTPase-activating protein (GAP) activity, affecting Rheb and TSC2 protein stability and resulting in mTORC1 activation [51]. Another essential regulator of mTORC1 is amino acids, the sensor of which is the Rag GTPase on the surface of lysosomes. Rag proteins recruit mTORC1 to lysosomes upon detection of amino acid sufficiency [52], and TSC suppresses mTORC1 upon amino acid deficiency [53]; these processes result in either positive or negative regulation (Figure 2).

The PI3K pathway is negatively regulated by several cellular machineries [54]. The tumor suppressor phosphatase and tensin homologue (PTEN) plays a role as an inhibitor by dephosphorylating PIP3 to convert it to PIP2 [55]. Insulin receptor substrate-1 (IRS-1), which is an adaptor protein of RTKs that provides the binding site for PI3K, is subject to negative regulation by PI3K through its degradation by proteasomes [56], decreased interaction with RTK due to the phosphorylation of IRS-1 by S6K, which is downstream of mTORC1 [57], and transcriptional suppression [58].

In a nutrient-replete state, an inhibitor of mTORC1 suppresses macropinocytosis. Bone marrow-derived dendritic cells are inhibited in performing macropinocytosis by rapamycin at a low, physiologically relevant concentration (1 ng/mL) [59]. In myeloid cells, growth factors, such as granulocyte/macrophage colony-stimulating factor (GM-CSF) and FMS-related tyrosine kinase 3 ligand (FLT3L), Toll-like receptors (TLRs) and cytokines, such as IL-4 and IL-15, promote mTOR activity, whereas the mTOR pathway is inactivated in the absence of these signals [60]. On the other hand, resting fibroblasts and other primary cells show the activation of mTOR even in the absence of extracellular signaling of PI3K. One report showed increased macropinocytosis in Ras-overexpressing cancerous cells even upon the addition of rapamycin [61]. During starvation, suppression of mTORC1 stimulates the degradation of proteins in lysosomes to enhance survival without affecting the macropinocytosis machinery [62]. In cells with constitutively active Ras under nutrient-replete conditions, activated mTORC1 directs protein synthesis and cell growth by utilizing extracellular amino acids and inhibits lysosomal catabolism of proteins engulfed via macropinocytosis [62]. On the other hand, in a nutrient-depleted environment, mTORC1 is suppressed, which results in the derepression of lysosomal catabolism. The derepression is the reason why hypovascular tumors can grow in a poor nutrient milieu. In cells with constitutively active Ras, mTORC1 does not seem to significantly regulate macropinocytosis under either nutrient-replete or nutrient-depleted conditions. Macropinocytosis is the molecular underpinning of exogenous mitochondrial transfer and shares its regulatory pathway with mTOR. Macropinocytosis is the molecular underpinning of exogenous mitochondrial transfer and shares its regulatory pathway with mTOR. Understanding these signaling cascades could be useful for developing a better mitochondrial transfer protocol.

## 3. Relations with Internalized Mitochondria and Endosomes

Following the internalization of mitochondria by macropinocytosis, the mitochondrial trajectory has not yet been revealed in detail. For entrapped viruses in endosomes, it is essential to break through the membranes of endosomes to the final destination. Viruses take advantage of various host machineries along with their own components. On the other hand, direct interaction between endogenous mitochondria and endosomes provides not only ion transport but also stress responses. In addition, some experiments showed that packaging of endogenous mitochondria allows them to be exported into extracellular spaces [63,64,65]. The examination of the endosomal escape of viruses and the exocytosis might provide useful clues as to how exogenous mitochondria dwell inside new host cells.

### 3.1. Endosomal Escapes for Viruses 

**Figure 3:**
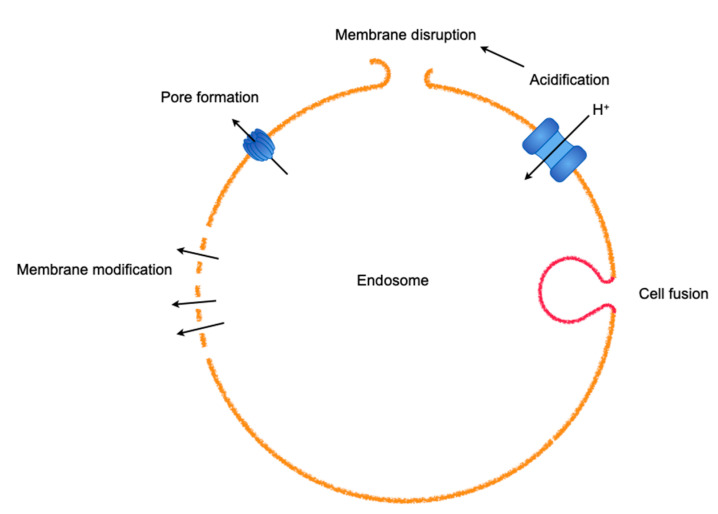
An array of methods used by viruses to escape from entrapment by endosomes. A virus often harnesses various machineries in combination.

Upon endosomal escape of viruses, reverse signaling from the host molecular machinery to the virus facilitates the release of the viral genome [29]. In nonacidic early-stage endosomes and macropinosomes containing incoming virions, since damaged adenoviruses expose protein VI to the plasma membrane upon cell entry, causing the membrane to be leaky [25], damaged virions in endosomes could be primed for endosomal escape via the leakage of membranes in concert with the fluxing of inorganic ions, such as sodium, potassium, chloride, or calcium, which are regulated by ion pumps and exchangers found in the endosomal membrane [66]. Serious disrupted endosomal membranes in the process of endosomal penetration are swiftly expelled by endosomophagy [25]. Unlike nonenveloped viruses such as adenoviruses, enveloped viruses such as orthomyxoviruses and rhabdoviruses make use of viral fusion proteins that are embedded in the viral envelopes [67]. The fusion proteins are activated by low pH, which is attributed to the vacuolar ATPase that functions to acidify intracellular compartments through the pumping of protons [68] and the rearrangement of viral proteins, such as Lassa virus glycoprotein [69], or the cleavage of components in the outermost layer, such as Ebola virus glycoprotein, by the endosomal proteases cathepsin L and B [70]; this eventually results in the fusion of two lipid bilayers via intracellular receptor switching to release the viral genome into the cytosol [71]. This acidification is also involved in the uncoating of influenza A virus by opening the M2 proton channel, acidifying the interior of the viral particles [72]. Depending upon the payload to be liberated into the cytosol, endosomes are ruptured by transient membrane modification, pore formation, and serious disruption (Figure 3).

The viral particles released from endosomes are transported toward the minus ends of microtubules near the centrosome and the nucleus across the densely packed cytosol [73]. Although the complexity of microtubule-dependent virion movements that are executed by contra-directional microtubule motors, which consist of plus-end-directed kinesin Kif5B and KLC1/2 and minus-end-directed dynein/dynamin, still remain to be elucidated [74], a stochastic model in which the directionality can be determined with certainty was proposed [75] that well explained the movement of peroxisomes in the cytosol [76]. During the translocation of virions, the nucleus exports the nuclear export factor CRM1 (chromosome region maintenance 1), which predominantly exists in the nucleus and exports a leucine-rich nuclear export sequence-containing protein upon the stimulation of Ran-GAP [77,78], consequently leading to detachment of the virion from the microtubules [79]. Adenoviruses exploit this dislocation to enable them access to the nuclear pore complex (NPC), which is a selective channel for small molecules less than 6 nm (40 kDa) spanning the nuclear envelope, and release their genomic contents to the final destination, the nucleus [80]. Whereas the host causes NPCs to link to microtubules, which allows the removal of virions from NPCs, the force applied to virions makes the capsid vulnerable, thus resulting in the destruction of the capsid and the liberation of the genome into the nucleus [81]. Such physical force in the cytosol might function in mitochondrial transfer to liberate the mitochondrial genome to the final destination.

### 3.2. Mitochondria–Endosome Interactions and Mitochondrial Exports 

**Figure 4:**
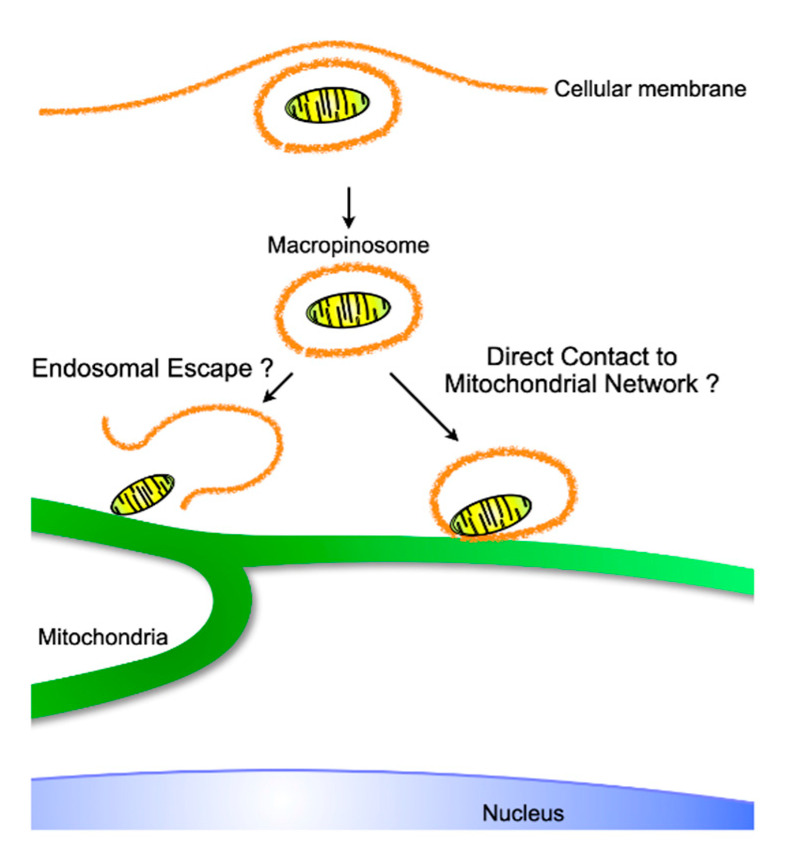
Putative intracellular trajectory of exogeneous internalized mitochondria, which originate from either isolated or exosome-derived mitochondria. Similar to viral endosomal escape, mitochondria might be liberated to make direct contact with the pre-existing mitochondrial network, or, as observed during iron transport, endosomes containing mitochondria might physiologically transport material originating from exogeneous mitochondria.

Mitochondria form microdomains when they are in contact with other intracellular organelles, such as the endoplasmic reticulum (ER), and exchange ions and metabolites essential for cellular homeostasis through mutual support [82]. Whereas the dynamic platform between the mitochondria and the ER is termed the mitochondria-associated membrane (MAM), which is fundamental for Ca^2+^ and glucose homeostasis, lipid trafficking, autophagy, apoptosis, and immunity [83,84,85], endosome–mitochondria interactions function as transport sites for iron and cholesterol, which are indispensable for the synthesis of iron-sulfur clusters and steroid hormones, respectively [86,87]. The binding occurs through VPS13A/13C [88], which regulates lipid metabolism [89], and MFN2 [90], which is in charge of mitochondrial fusion and MAM [91]. As MAMs are involved in innate immunity, ROS are released into endosomes containing *Staphylococcus aureus* through contact with mitochondria-derived vesicles (MDVs) to kill the bacteria [92]. Moreover, mitochondria are recruited to the parasitophorous vacuole membrane (PVM) that holds parasites such as *Toxoplasma gondii*, which are deprived of nutrients by mitochondria, resulting in starvation of the parasites [93]. Thus, these microdomains between endosomes and mitochondria open up to exchange materials ranging from ions to sizable biomolecules. The formation and export of MDVs containing selective mitochondrial contents, such as damaged mitochondria, were reported in the immune system [64] and central neural system [65]. Furthermore, a report that the export of whole mitochondria by healthy cells can rescue growth in respiration-deficient tumors [63] might suggest the existence of a machinery that packages endogenous pinched-off mitochondria in mitochondrial networks, which might result in their entrapment for export rather than their digestion by mitophagy [94]. Given that there is a packing mechanism, the cargo unpacking or leakage machinery might exist, and thus lead to the engraftment of exogeneous mitochondria in the cytosol, although this requires more investigation. Concern has arisen about the intactness of exogenous mitochondria in the extracellular space or endosomes [95]. Even at micromolar concentrations of Ca^2+^ in the medium, influx into matrices through the permeability transition pore is nonselectively effective for molecules with molecular masses of up to 1600 kDa [96]. Subsequent swelling of the mitochondria to equalize the osmolarity of the mitochondrial matrices and the medium could lead to irreversible destruction of the mitochondrial membrane. On the other hand, independent research has shown functional recovery of mitochondrial respiration with exogenous mitochondrial transfer into respiration-deficient cells. To resolve these discrepancies, I suppose that exogenous mitochondria function as a vehicle for mitochondrial DNA as cargo rather than functioning as respiratory units. Either endosomal escape of exogenous mitochondria into the cytoplasm or direct contact of endosome-enclosed exogenous mitochondria with endogenous mitochondrial networks might deliver the mitochondrial DNAs to endogenous mitochondria, resulting in rescue of respiratory function (Figure 4). When either exogenous mitochondria or mitochondrial DNA could be integrated into endogenous mitochondria, the function of endogenous mitochondria, including mitochondrial respiration, expression of mitochondria dynamics-related proteins, and mitochondrial ROS as signaling molecules, should affect the consequences.

## 4. Artificial Mitochondrial Transfer

Since mitochondrial transfer has been reported and shown to function in pathophysiological conditions, interventions using mitochondrial transfer to treat various diseases have been investigated intensively [15,97,98,99], and some protocols have reached the clinical application stage [100,101]. A challenge for this process is the poor efficiency of the transfer, which might weaken the effects on the disease, despite some parameters such as ATP production and mitochondrial ROS generation being improved to some degree. In recent years, various methods have been developed to artificially introduce isolated exogenous mitochondria into cells (Table 2). These methods, using cell penetrating peptides (CPPs) [7], centrifugal force [8], magnetic force [9], and vapor bubbles [10], successfully and more efficiently internalized exogenous mitochondria into cells.

### 4.1. Cell Penetrating Peptide

Maeda et al. designed the Trans-Activator of Transcription protein (TAT) conjugated with dextran to enable complex formation and isolated mitochondria with TAT-dextran, which allowed easier penetration of the plasma membrane through the reduction of the net negative charges, and the properties of CPP and TAT, independent of macropinocytosis, demonstrated that the complex can more efficiently transfer exogenous mitochondria into cells [7]. The improvement of the transfer efficiency was verified by using primary rat neonatal cardiomyocytes that were exposed to oxidative stress based on the survival and the reduced apoptosis signals following stress. As viruses utilize independent pleural machinery to enter cells, exogeneous mitochondria might take advantage of forms of endocytosis other than macropinocytosis, through which isolated exogeneous mitochondria are mainly engulfed by simple in vitro coincubation [4].

### 4.2. Centrifugal Force

A simple and easy mitochondrial transfer protocol using centrifugation was reported in various types of cells, including cancer cell lines and mesenchymal stem cells [8]. The protocol showed better transfer efficiency in the examined cell types, as the TAT-dextran protocol improved the transfer efficiency. The authors found that mitochondrial transfer significantly increased ATP production, the membrane potential, and the basal oxygen consumption rate in cells that received exogeneous mitochondria. The system is simple and rapid for introducing mitochondria and may be adaptable not only to adherent cells but also to floating cells. The mode of action of centrifugation could be to localize cells and mitochondria in close proximity. The activation of macropinocytosis through the mTORC pathway could be feasible in combination with this protocol to obtain improved efficiency, although it is not as simple to regulate macropinocytosis as described above. The aberrant activation of macropinocytosis is implicated in cancer [102], neurogenerative diseases [103], and atherosclerosis [104].

### 4.3. Magnetic Force Using Anti-Mitochondria Specific Protein with Magnetic Nanoparticles

Instead of centrifugation force, magnetic force was applied to develop a new protocol for mitochondrial transfer by increasing the contiguity of cells [9]. In this protocol, TOM22, which is a mitochondrial import receptor along with TOM20 and TOM40 on the mitochondrial outer membrane, was used as a tether for binding with microbead-conjugated antibodies. This method was also more efficient in transferring mitochondria into cells with respect to functionality, similar to the TAT-dextran and centrifugation protocol. There is a concern that the magnetic beads may remain in cells after mitochondrial transplantation after 4 days, and it is unclear how these magnetic beads affect cellular function.

### 4.4. Generation of Vapor Bubbles by a Photothermal Nanoblade

Photothermal nanoblades are metal nanostructures that generate highly localized and shaped explosive cavitation bubbles by light energy derived from laser pulses. These bubbles are capable of temporally disturbing the cell membranes. A protocol using this blade to perforate the cell membrane and transfer isolated mitochondria into cells was developed [10]. The authors reported that mitochondrial transfer restored the respiratory capacity of mitochondria-deficient cells and that the metabolic-related gene expression patterns and metabolic profiles were also close to those of the original cells. Although this technique is unsuitable for mitochondrial transfer into many types of cells, it might be an alternative to direct injection for eggs.

## 5. Perspective

Current protocols for mitochondrial transfer focus on the first step, which involves crossing the plasma membrane, whereas endosomal escape could be an essential step required for exogeneous mitochondria to function in new hosts and could be the next target in further investigations. In addition to internalization and endosomal escape, another issue is the preparation of isolated mitochondria for clinical intervention using mitochondrial transfer. When significantly damaged mitochondria are transferred into the cell, two distinct fates other than stable engraftment could occur: digestion by mitophagy, which might outsource this process [64], and provocation of the innate immune response by mtDNA and various proteins released from damaged mitochondria as DAMPs [105]. Considering that viruses utilize damage that occurs upon crossing the plasma membrane to escape endosomes, it might be not necessary to ensure the intactness of isolated mitochondria too strictly if the mitochondrial genome is safely protected from any damage.

Improved understanding of the trajectory of exogenous mitochondria from cell entry to engraftment in the host cytosol could shed light on not only the evolutionary process of symbiosis but also new aspects of the development of a novel, enhanced mitochondrial transfer method to treat diseases involving mitochondrial dysfunction.

## Figures and Tables

**Table 1:** Exogenous mitochondrial transplantation in animal models.

Target	Diseases	Method	Outcome	Animal	Ref./Year
Brain	Ischemia reperfusion injury	Direct injection	Enhanced survival-related gene expression	Mice	[15]/2016
Brain	Ischemia reperfusion injury	Intra-arterial injection	Functional recovery	Rats	[16]/2016
Spine	Spinal cord injury	Direct injection	Improved mitochondrial respiration	Rats	[17]/2018
Heart	Ischemia reperfusion injury	Direct injection	Enhanced post-infarct cardiac function	Rabbits	[18]/2013
Liver	Ischemia reperfusion injury	Direct injection into the spleen	Suppressed necrosis and apoptosis	Rats	[19]/2013
Lung	Ischemia reperfusion injury	Intra-arterial or trans-tracheal injection	Increased compliance and inspiratory capacity	Mice	[20]/2020

**Table 2:** Current reported technologies used to enhance mitochondrial transfer in vitro.

Method		Recipient Cell		Donor Mitochondria	Mitochondria Marker	Ref./Year
TAT-dextran	Human	Uterine endometrial gland-derived mesenchymal cells	Rat	C2C12, an immortalized myoblast cell line	MitoDsRed	[7]/2020
	Rat	Neonate cardiomyocytes, primary	Rat	C2C12, an immortalized myoblast cell line	MitoDsRed	
Centrifugation	Rat	L6, muscle	Human	Umbilical cord-derived mesenchymal stem cells	MitoTracker	[8]/2018
Magnetomitotransfer	Human	MRC-5, fibroblasts	Human	MRC-5, a diploid human cell culture line composed of fibroblasts, originally developed from research deriving lung tissue of a 14 week old aborted Caucasian male fetus	MitoTracker	[9]/2016
Photothermal nanoblade	Human	143BTK-, osteosarcoma	Human	MDA-MB-453, breast carcinoma	MitoDsRed	[10]/2016

## Abbreviations

CAR	Coxsackievirus adenovirus receptor
VSAP	Vasodilator-stimulated phosphoprotein
PIP3	Phosphatidylinositol (3,4,5)-triphosphate
RTKs	Receptor tyrosine kinases
M-CSF	Macrophage colony-stimulating factor
PH	Pleckstrin homology
PLCγ	Phospholipase C-γ
IP3	Inositol 1,4,5-trisphosphate
DAG	Diacylglycerol
PKC	Protein kinase C
mTOR	Mechanistic target of rapamycin
MAP	Mitogen-activated protein
PIP2	Phosphatidylinositol (4,5)-bisphosphate
TSC2	Tuberous sclerosis complex 2
Rheb	Ras homolog enriched in the brain
GAP	GTPase-activating protein
PTEN	Phosphatase and tensin homologue
IRS-1	Insulin receptor substrate-1
GM-CSF	Granulocyte/macrophage colony-stimulating factor
FLT3L	FMS-related tyrosine kinase 3 ligand
TLRs	Toll-like receptors
CRM1	Chromosome region maintenance 1
NPC	Nuclear pore complex
ER	Endoplasmic reticulum
MAM	Mitochondria-associated membrane
MDVs	Mitochondria-derived vesicles
PVM	Parasitophorous vacuole membrane
CPPs	Cell penetrating peptides
TAT	Trans-Activator of Transcription protein

## References

[1] Lane N. (2014). Bioenergetic Constraints on the Evolution of Complex Life. Cold Spring Harb. Perspect. Boil..

[2] Wallace D.C., Chalkia D. (2013). Mitochondrial DNA Genetics and the Heteroplasmy Conundrum in Evolution and Disease. Cold Spring Harb. Perspect. Boil..

[3] De Carvalho T.M.U., Barrias E.S., De Souza W. (2015). Macropinocytosis: a pathway to protozoan infection. Front. Physiol..

[4] Kitani T., Kami D., Matoba S., Gojo S. (2014). Internalization of isolated functional mitochondria: involvement of macropinocytosis. J. Cell. Mol. Med..

[5] Yoshida S., Pacitto R., Inoki K., Swanson J.A. (2017). Macropinocytosis, mTORC1 and cellular growth control. Cell. Mol. Life Sci..

[6] Spees J.L., Olson S.D., Whitney M.J., Prockop D.J. (2006). Mitochondrial transfer between cells can rescue aerobic respiration. Proc. Natl. Acad. Sci. USA.

[7] Maeda H., Kami D., Maeda R., Murata Y., Jo J.-I., Kitani T., Tabata Y., Matoba S., Gojo S. (2020). TAT-dextran–mediated mitochondrial transfer enhances recovery from models of reperfusion injury in cultured cardiomyocytes. J. Cell. Mol. Med..

[8] Kim M.J., Hwang J.W., Yun C.-K., Lee Y., Choi Y.-S. (2018). Delivery of exogenous mitochondria via centrifugation enhances cellular metabolic function. Sci. Rep..

[9] Macheiner T., Fengler V.H.I., Agreiter M., Eisenberg T., Madeo F., Kolb D., Huppertz B., Ackbar R., Sargsyan K. (2016). Magnetomitotransfer: An efficient way for direct mitochondria transfer into cultured human cells. Sci. Rep..

[10] Wu T.-H., Sagullo E., Case D., Zheng X., Li Y., Hong J.S., TeSlaa T., Patananan A.N., McCaffery J.M., Niazi K. (2016). Mitochondrial Transfer by Photothermal Nanoblade Restores Metabolite Profile in Mammalian Cells. Cell Metab..

[11] Rustom A., Saffrich R., Walther P., Marković I., Gerdes H.-H. (2004). Nanotubular Highways for Intercellular Organelle Transport. Science.

[12] Greenfield A., Braude P., Flinter F., Lovell-Badge R., Ogilvie C., Perry A.C.F. (2017). Assisted reproductive technologies to prevent human mitochondrial disease transmission. Nat. Biotechnol..

[13] Tan A.S., Baty J.W., Dong L., Bezawork-Geleta A., Endaya B., Goodwin J., Bajzikova M., Kovarova J., Peterka M., Yan B. (2015). Mitochondrial Genome Acquisition Restores Respiratory Function and Tumorigenic Potential of Cancer Cells without Mitochondrial DNA. Cell Metab..

[14] Valdinocci D., Simões R., Kovarova J., Cunha-Oliveira T., Neuzil J., Pountney D. (2019). Intracellular and Intercellular Mitochondrial Dynamics in Parkinson’s Disease. Front. Mol. Neurosci..

[15] Hayakawa K., Esposito E., Wang X., Terasaki Y., Liu Y., Xing C., Ji X., Lo E.H. (2016). Transfer of mitochondria from astrocytes to neurons after stroke. Nature.

[16] Huang P.-J., Kuo C.-C., Lee H.-C., Shen C.-I., Cheng F.-C., Wu S.-F., Chang J.C., Pan H.-C., Lin S.-Z., Liu C.-S. (2016). Transferring Xenogenic Mitochondria Provides Neural Protection against Ischemic Stress in Ischemic Rat Brains. Cell Transplant..

[17] Gollihue J.L., Patel S.P., Eldahan K.C., Cox D.H., Donahue R., Taylor B., Sullivan P.G., Rabchevsky A.G. (2018). Effects of Mitochondrial Transplantation on Bioenergetics, Cellular Incorporation, and Functional Recovery after Spinal Cord Injury. J. Neurotrauma.

[18] Masuzawa A., Black K.M., Pacak C.A., Ericsson M., Barnett R.J., Drumm C., Seth P., Bloch D.B., Levitsky S., Cowan D.B. (2013). Transplantation of autologously derived mitochondria protects the heart from ischemia-reperfusion injury. Am. J. Physiol. Circ. Physiol..

[19] Lin H.-C., Liu S.-Y., Lai H.-S., Lai I.-R. (2013). Isolated Mitochondria Infusion Mitigates Ischemia-Reperfusion Injury of the Liver in Rats. Shock.

[20] Moskowitzova K., Orfany A., Liu K., Ramirez-Barbieri G., Thedsanamoorthy J.K., Yao R., Guariento A., Doulamis I.P., Blitzer D., Shin B. (2019). Mitochondrial transplantation enhances murine lung viability and recovery after ischemia-reperfusion injury. Am. J. Physiol. Cell. Mol. Physiol..

[21] Bloomfield G., Kay R.R. (2016). Uses and abuses of macropinocytosis. J. Cell Sci..

[22] Mercer J., Helenius A. (2009). Virus entry by macropinocytosis. Nature.

[23] Helmuth J.A., Burckhardt C.J., Koumoutsakos P., Greber U.F., Sbalzarini I. (2007). A novel supervised trajectory segmentation algorithm identifies distinct types of human adenovirus motion in host cells. J. Struct. Boil..

[24] Greber U.F., Flatt J.W. (2019). Adenovirus Entry: From Infection to Immunity. Annu. Rev. Virol..

[25] Luisoni S., Suomalainen M., Boucke K., Tanner L.B., Wenk M.R., Guan X.L., Grzybek M., Coskun Ü., Greber U.F. (2015). Co-option of Membrane Wounding Enables Virus Penetration into Cells. Cell Host Microbe.

[26] Burckhardt C.J., Greber U.F. (2009). Virus Movements on the Plasma Membrane Support Infection and Transmission between Cells. PLoS Pathog..

[27] Pache L., Venkataraman S., Reddy V.S., Nemerow G. (2008). Structural Variations in Species B Adenovirus Fibers Impact CD46 Association. J. Virol..

[28] Amstutz B., Gastaldelli M., Kälin S., Imelli N., Boucke K., Wandeler E., Mercer J., Hemmi S., Greber U.F. (2008). Subversion of CtBP1-controlled macropinocytosis by human adenovirus serotype 3. EMBO J..

[29] Wolfrum N., Greber U.F. (2012). Adenovirus signalling in entry. Cell. Microbiol..

[30] Kälin S., Amstutz B., Gastaldelli M., Wolfrum N., Boucke K., Havenga M., Digennaro F., Liska N., Hemmi S., Greber U.F. (2010). Macropinocytotic Uptake and Infection of Human Epithelial Cells with Species B2 Adenovirus Type 35. J. Virol..

[31] Liberali P., Kakkonen E., Turacchio G., Valente C., Spaar A., Perinetti G., Bockmann R.A., Corda D., Colanzi A., Marjomaki V. (2008). The closure of Pak1-dependent macropinosomes requires the phosphorylation of CtBP1/BARS. EMBO J..

[32] Mercer J., Helenius A. (2008). Vaccinia Virus Uses Macropinocytosis and Apoptotic Mimicry to Enter Host Cells. Science.

[33] Niebuhr K., Giuriato S., Pedron T., Philpott D.J., Gaits-Iacovoni F., Sable J., Sheetz M.P., Parsot C., Sansonetti P.J., Payrastre B. (2002). Conversion of PtdIns(4,5)P2 into PtdIns(5)P by the S.flexneri effector IpgD reorganizes host cell morphology. EMBO J..

[34] Moser T.S., Jones R.G., Thompson C.B., Coyne C.B., Cherry S. (2010). A Kinome RNAi Screen Identified AMPK as Promoting Poxvirus Entry through the Control of Actin Dynamics. PLoS Pathog..

[35] Kondratowicz A.S., Hunt C.L., Davey R.A., Cherry S., Maury W. (2012). AMP-Activated Protein Kinase Is Required for the Macropinocytic Internalization of Ebolavirus. J. Virol..

[36] Albert M.L. (2004). Death-defying immunity: do apoptotic cells influence antigen processing and presentation?. Nat. Rev. Immunol..

[37] González A., Hall M.N. (2017). Nutrient sensing and TOR signaling in yeast and mammals. EMBO J..

[38] Yoshida S., Hoppe A.D., Araki N., Swanson J.A. (2009). Sequential signaling in plasma-membrane domains during macropinosome formation in macrophages. J. Cell Sci..

[39] Chen C.-L., Wang Y., Sesaki H., Iijima M. (2012). Myosin I Links PIP3 Signaling to Remodeling of the Actin Cytoskeleton in Chemotaxis. Sci. Signal..

[40] Vanhaesebroeck B., Stephens L.R., Hawkins P. (2012). PI3K signalling: the path to discovery and understanding. Nat. Rev. Mol. Cell Boil..

[41] Azzi A., Boscoboinik D., Hensey C. (1992). The protein kinase C family. JBIC J. Boil. Inorg. Chem..

[42] Apgar J.R. (1995). Activation of protein kinase C in rat basophilic leukemia cells stimulates increased production of phosphatidylinositol 4-phosphate and phosphatidylinositol 4,5-bisphosphate: correlation with actin polymerization. Mol. Boil. Cell.

[43] Saxton R.A., Sabatini D.M. (2017). mTOR Signaling in Growth, Metabolism, and Disease. Cell.

[44] Nussinov R., Tsai C.-J., Jang H. (2019). Is Nanoclustering essential for all oncogenic KRas pathways? Can it explain why wild-type KRas can inhibit its oncogenic variant?. Semin. Cancer Boil..

[45] Nussinov R., Tsai C.-J., Jang H. (2019). Does Ras Activate Raf and PI3K Allosterically?. Front. Oncol..

[46] Yang G., Murashige D.S., Humphrey S.J., James D.E. (2015). A Positive Feedback Loop between Akt and mTORC2 via SIN1 Phosphorylation. Cell Rep..

[47] Ma L., Chen Z., Erdjument-Bromage H., Tempst P., Pandolfi P.P. (2005). Phosphorylation and Functional Inactivation of TSC2 by Erk. Cell.

[48] Inoki K., Li Y., Zhu T., Wu J., Guan K.-L. (2002). TSC2 is phosphorylated and inhibited by Akt and suppresses mTOR signalling. Nature.

[49] Dibble C., Elis W., Menon S., Qin W., Klekota J., Asara J.M., Finan P.M., Kwiatkowski D.J., Murphy L.O., Manning B.D. (2012). TBC1D7 is a third subunit of the TSC1-TSC2 complex upstream of mTORC1. Mol. Cell.

[50] Betz C., Hall M.N. (2013). Where is mTOR and what is it doing there?. J. Cell Boil..

[51] Menon S., Dibble C., Talbott G., Hoxhaj G., Valvezan A.J., Takahashi H., Cantley L.C., Manning B.D. (2014). Spatial control of the TSC complex integrates insulin and nutrient regulation of mTORC1 at the lysosome. Cell.

[52] Bar-Peled L., Schweitzer L.D., Zoncu R., Sabatini D.M. (2012). Ragulator Is a GEF for the Rag GTPases that Signal Amino Acid Levels to mTORC1. Cell.

[53] Demetriades C., Doumpas N., A Teleman A. (2014). Regulation of TORC1 in response to amino acid starvation via lysosomal recruitment of TSC2. Cell.

[54] Harrington L.S., Findlay G.M., Lamb R.F. (2005). Restraining PI3K: mTOR signalling goes back to the membrane. Trends Biochem. Sci..

[55] Maehama T., Dixon J.E. (1998). The tumor suppressor, PTEN/MMAC1, dephosphorylates the lipid second messenger, phosphatidylinositol 3,4,5-trisphosphate. J. Boil. Chem..

[56] Kim S.J., DeStefano M.A., Oh W.J., Wu C.-C., Vega-Cotto N.M., Finlan M., Liu U., Su B., Jacinto E. (2012). mTOR complex 2 regulates proper turnover of insulin receptor substrate-1 via the ubiquitin ligase subunit Fbw8. Mol. Cell.

[57] Harrington L.S., Findlay G.M., Gray A., Tolkacheva T., Wigfield S., Rebholz H., Barnett J., Leslie N.R., Cheng S., Shepherd P.R. (2004). The TSC1-2 tumor suppressor controls insulin–PI3K signaling via regulation of IRS proteins. J. Cell Boil..

[58] Shah O.J., Wang Z., Hunter T. (2004). Inappropriate activation of the TSC/Rheb/mTOR/S6K cassette induces IRS1/2 depletion, insulin resistance, and cell survival deficiencies. Curr. Biol..

[59] Hackstein H., Taner T., Logar A.J., Thomson A.W. (2002). Rapamycin inhibits macropinocytosis and mannose receptor–mediated endocytosis by bone marrow–derived dendritic cells. Blood.

[60] Weichhart T., Hengstschläger M., Linke M. (2015). Regulation of innate immune cell function by mTOR. Nat. Rev. Immunol..

[61] Sung S., Choi J., Cheong H. (2015). Catabolic pathways regulated by mTORC1 are pivotal for survival and growth of cancer cells expressing mutant Ras. Oncotarget.

[62] Palm W., Park Y., Wright K., Pavlova N., Tuveson D.A., Thompson C.B. (2015). The Utilization of Extracellular Proteins as Nutrients Is Suppressed by mTORC1. Cell.

[63] Dong L., Kovarova J., Bajzikova M., Bezawork-Geleta A., Svec D., Endaya B., Sachaphibulkij K., Coelho A., Sebkova N., Ruzickova A. (2017). Horizontal transfer of whole mitochondria restores tumorigenic potential in mitochondrial DNA-deficient cancer cells. eLife.

[64] Phinney D., Di Giuseppe M., Njah J., Sala-Llinas E., Shiva S., Croix C.M.S., Stolz N.B., Watkins S.C., Di Y., Leikauf G.D. (2015). Mesenchymal stem cells use extracellular vesicles to outsource mitophagy and shuttle microRNAs. Nat. Commun..

[65] Davis C.-H.O., Kim K.-Y., Bushong E.A., Mills E.A., Boassa D., Shih T., Kinebuchi M., Phan S., Zhou Y., Bihlmeyer N. (2014). Transcellular degradation of axonal mitochondria. Proc. Natl. Acad. Sci. USA.

[66] Seth P., Pastan I., Willingham M.C. (1987). Adenovirus-dependent changes in cell membrane permeability: role of Na+, K+-ATPase. J. Virol..

[67] Harrison S.C. (2015). Viral membrane fusion. Virology.

[68] Nelson N., Taiz L. (1989). The evolution of H+-ATPases. Trends Biochem. Sci..

[69] Jae L.T., Raaben M., Riemersma M., Van Beusekom E., Blomen V.A., Velds A., Kerkhoven R.M., Carette J.E., Topaloglu H., Meinecke P. (2013). Deciphering the Glycosylome of Dystroglycanopathies Using Haploid Screens for Lassa Virus Entry. Science.

[70] Brecher M., Schornberg K.L., Delos S.E., Fusco M.L., Saphire E.O., White J.M. (2011). Cathepsin Cleavage Potentiates the Ebola Virus Glycoprotein To Undergo a Subsequent Fusion-Relevant Conformational Change. J. Virol..

[71] Staring J., Raaben M., Brummelkamp T.R. (2018). Viral escape from endosomes and host detection at a glance. J. Cell Sci..

[72] Cady S.D., Luo W., Hu F., Hong M. (2009). Structure and Function of the Influenza A M2 Proton Channel. Biochemistry.

[73] Leopold P.L., Kreitzer G., Miyazawa N., Rempel S., Pfister K.K., Rodriguez-Boulan E., Crystal R.G. (2000). Dynein- and Microtubule-Mediated Translocation of Adenovirus Serotype 5 Occurs after Endosomal Lysis. Hum. Gene Ther..

[74] Dodding M.P., Way M. (2011). Coupling viruses to dynein and kinesin-1. EMBO J..

[75] Gazzola M., Burckhardt C.J., Bayati B., Engelke M.F., Greber U.F., Koumoutsakos P. (2009). A Stochastic Model for Microtubule Motors Describes the In Vivo Cytoplasmic Transport of Human Adenovirus. PLoS Comput. Boil..

[76] Kural C., Kim H., Syed S., Goshima G., Gelfand V.I., Selvin P.R. (2005). Kinesin and Dynein Move a Peroxisome in Vivo: A Tug-of-War or Coordinated Movement?. Science.

[77] Fornerod M., Ohno M., Yoshida M., Mattaj I. (1997). CRM1 Is an Export Receptor for Leucine-Rich Nuclear Export Signals. Cell.

[78] Mahajan R., Delphin C., Guan T., Gerace L., Melchior F. (1997). A Small Ubiquitin-Related Polypeptide Involved in Targeting RanGAP1 to Nuclear Pore Complex Protein RanBP2. Cell.

[79] Strunze S., Trotman L.C., Boucke K., Greber U.F. (2005). Nuclear Targeting of Adenovirus Type 2 Requires CRM1-mediated Nuclear Export. Mol. Boil. Cell.

[80] Trotman L.C., Mosberger N., Fornerod M., Stidwill R.P., Greber U.F. (2001). Import of adenovirus DNA involves the nuclear pore complex receptor CAN/Nup214 and histone H1. Nat. Cell Biol..

[81] Jahed Z., Soheilypour M., Peyro M., Mofrad M.R.K. (2016). The LINC and NPC relationship - it’s complicated!. J. Cell Sci..

[82] Daniele T., Schiaffino M.V. (2014). Organelle biogenesis and interorganellar connections: Better in contact than in isolation. Commun. Integr. Boil..

[83] Szymański J., Janikiewicz J., Michalska B., Patalas-Krawczyk P., Perrone M., Ziółkowski W., Duszynski J., Pinton P., Dobrzyń A., Wieckowski M.R. (2017). Interaction of Mitochondria with the Endoplasmic Reticulum and Plasma Membrane in Calcium Homeostasis, Lipid Trafficking and Mitochondrial Structure. Int. J. Mol. Sci..

[84] Rieusset J. (2018). The role of endoplasmic reticulum-mitochondria contact sites in the control of glucose homeostasis: an update. Cell Death Dis..

[85] Martinvalet D. (2018). The role of the mitochondria and the endoplasmic reticulum contact sites in the development of the immune responses. Cell Death Dis..

[86] Sheftel A.D., Zhang A.-S., Brown C.M., Shirihai O.S., Ponka P. (2007). Direct interorganellar transfer of iron from endosome to mitochondrion. Blood.

[87] Charman M., Kennedy B.E., Osborne N., Karten B. (2009). MLN64 mediates egress of cholesterol from endosomes to mitochondria in the absence of functional Niemann-Pick Type C1 protein. J. Lipid Res..

[88] Kumar N., Leonzino M., Hancock-Cerutti W., Horenkamp F.A., Li P., Lees J.A., Wheeler H., Reinisch K.M., De Camilli P. (2018). VPS13A and VPS13C are lipid transport proteins differentially localized at ER contact sites. J. Cell Boil..

[89] Park J.-S., Thorsness M.K., Policastro R., McGoldrick L.L., Hollingsworth N.M., Thorsness P.E., Neiman A.M. (2016). Yeast Vps13 promotes mitochondrial function and is localized at membrane contact sites. Mol. Boil. Cell.

[90] Daniele T., Hurbain I., Vago R., Casari G., Raposo G., Tacchetti C., Schiaffino M.V. (2014). Mitochondria and Melanosomes Establish Physical Contacts Modulated by Mfn2 and Involved in Organelle Biogenesis. Curr. Boil..

[91] Naon D.P., Zaninello M., Giacomello M., Varanita T., Grespi F., Lakshminaranayan S., Serafini A., Semenzato M., Herkenne S., Hernandez-Alvarez M.I. (2016). Critical reappraisal confirms that Mitofusin 2 is an endoplasmic reticulum-mitochondria tether. Proc. Natl. Acad. Sci. USA.

[92] Abuaita B.H., Schultz T.L., O’Riordan M. (2018). Mitochondria-Derived Vesicles Deliver Antimicrobial Reactive Oxygen Species to Control Phagosome-Localized Staphylococcus aureus. Cell Host Microbe.

[93] Pernas L., Bean C., Boothroyd J.C., Scorrano L. (2018). Mitochondria Restrict Growth of the Intracellular Parasite Toxoplasma gondii by Limiting Its Uptake of Fatty Acids. Cell Metab..

[94] Todkar K., Chikhi L., Germain M. (2019). Mitochondrial interaction with the endosomal compartment in endocytosis and mitochondrial transfer. Mitochondrion.

[95] Chernyak B.V. (2020). Mitochondrial Transplantation: A Critical Analysis. Biochemistry (Mosc.).

[96] Bernardi P., Rasola A., Forte M., Lippe G. (2015). The Mitochondrial Permeability Transition Pore: Channel Formation by F-ATP Synthase, Integration in Signal Transduction, and Role in Pathophysiology. Physiol. Rev..

[97] Marlein C.R., Zaitseva L., Piddock R.E., Robinson S.D., Edwards D.R., Shafat M.S., Zhou Z., Lawes M., Bowles K.M., Rushworth S.A. (2017). NADPH oxidase-2 derived superoxide drives mitochondrial transfer from bone marrow stromal cells to leukemic blasts. Blood.

[98] Pour P.A., Kenney M.C., Kheradvar A. (2020). Bioenergetics Consequences of Mitochondrial Transplantation in Cardiomyocytes. J. Am. Hear. Assoc..

[99] Sinha P., Islam M.N., Bhattacharya S., Bhattacharya J. (2016). Intercellular mitochondrial transfer: bioenergetic crosstalk between cells. Curr. Opin. Genet. Dev..

[100] Chang C.-Y., Liang M.-Z., Chen L. (2019). Current progress of mitochondrial transplantation that promotes neuronal regeneration. Transl. Neurodegener..

[101] Emani S.M., McCully J.D. (2018). Mitochondrial transplantation: applications for pediatric patients with congenital heart disease. Transl. Pediatr..

[102] Commisso C., Davidson S.M., Soydaner-Azeloglu R.G., Parker S.J., Kamphorst J.J., Hackett S., Grabocka E., Nofal M., Drebin J.A., Thompson C.B. (2013). Macropinocytosis of protein is an amino acid supply route in Ras-transformed cells. Nature.

[103] von der Haar T., Josse L., Wright P., Zenthon J., Tuite M.F. (2007). Development of a novel yeast cell-based system for studying the aggregation of Alzheimer’s disease-associated Abeta peptides in vivo. Neurodegener Dis..

[104] Kruth H.S., Jones N.L., Huang W., Zhao B., Ishii I., Chang J., Combs C.A., Malide D., Zhang W.-Y. (2004). Macropinocytosis Is the Endocytic Pathway That Mediates Macrophage Foam Cell Formation with Native Low 105 Density Lipoprotein. J. Boil. Chem..

[105] West A.P., Shadel G.S., Ghosh S. (2011). Mitochondria in innate immune responses. Nat. Rev. Immunol..

